# Identification of novel prognostic biomarkers in the TF-enhancer-target regulatory network in hepatocellular carcinoma and immune infiltration analysis

**DOI:** 10.3389/fgene.2023.1158341

**Published:** 2023-03-29

**Authors:** Jianing Yan, Guoliang Ye, Yongfu Shao, Hanxuan Zhou

**Affiliations:** ^1^ Department of Gastroenterology, The First Affiliated Hospital of Ningbo University, Ningbo, China; ^2^ Department of Gastroenterology, Institute of Digestive Disease of Ningbo University, Ningbo, China; ^3^ Department of Pharmacy, Yinzhou Integrated TCM and Western Medicine Hospital, Ningbo, China

**Keywords:** hepatocellular carcinoma, DAPk1, eRNA, enhancer, prognosis, regulatory network

## Abstract

**Background:** Hepatocellular carcinoma (HCC) remains notorious for its high malignancy, poor prognosis and high mortality. The exploration of novel therapeutic agents for HCC has remained challenging due to its complex aetiology. Therefore, it is necessary to elucidate the pathogenesis and mechanism of HCC for clinical intervention.

**Methods:** We collected data from several public data portals and systematically analysed the association between transcription factors (TFs), eRNA-associated enhancers and downstream targets. We next filtered the prognostic genes and established a novel prognosis-related nomogram model. Moreover, we explored the potential mechanisms of the identified prognostic genes. The expression level was validated by several ways.

**Results:** We first constructed a significant TF-enhancer-target regulatory network and identified DAPK1 as a coregulatory differentially expressed prognosis-related gene. We combined common clinicopathological factors and built a prognostic nomogram model for HCC. We found that our regulatory network was correlated with the processes of synthesizing various substances. Moreover, we explored the role of DAPK1 in HCC and found that it was associated with immune cell infiltration and DNA methylation. Several immunostimulators and targeting drugs could be promising immune therapy targets. The tumor immune microenvironment was analyzed. Finally, the lower DAPK1 expression in HCC was validated *via* the GEO database, UALCAN cohort, and qRT-PCR.

**Conclusion:** In conclusion, we established a significant TF-enhancer-target regulatory network and identified downregulated DAPK1 as an important prognostic and diagnostic gene in HCC. Its potential biological functions and mechanisms were annotated using bioinformatics tools.

## 1 Introduction

Currently, hepatocellular carcinoma (HCC) is the leading cause of cancer-related mortality and the second most lethal malignancy among gastrointestinal malignant tumours worldwide (Sung et al., 2021). Immunotherapy now is experiencing its “golden age”, but the clinical long-term survival time of HCC patients remains unsatisfactory despite the breakthroughs made recently (Abushukair and Saeed, 2022; Zhao et al., 2022a). The unclear molecular biological mechanisms and pathogenesis of HCC are the major causes of chemoradiotherapy and immunotherapy failure, leading to a poor prognosis of HCC patients (Llovet et al., 2022). Therefore, it is urgent to investigate and explore the molecular biological mechanisms and underlying key genes of HCC carcinogenesis and progression.

The expression of many important genes is mainly controlled by cis-acting enhancers bound by transcription factors (TFs) (Lin et al., 2022). Enhancers can control transcriptional regulation by recruiting DNA-binding TFs in a tissue-specific manner in malignant tumours. Various TFs and cofactors combine with enhancers to assemble active enhancers, further inducing the expression of downstream mRNAs, long non-coding RNAs (lncRNAs) and microRNAs (miRNAs) (Marzi et al., 2016; Carullo et al., 2020). Recently, an increasing number of studies have found that enhancers can be transcribed into a novel kind of lncRNA defined as enhancer RNAs (eRNAs) that significantly regulate gene expression and downstream signalling pathways in malignant tumours, which reveals the functional diversity of enhancers (Fang et al., 2020; Sartorelli and Lauberth, 2020; Gao and Rong, 2022). However, until now, the relationships of only a minority of these molecules in HCC have been revealed. In general, it is essential to elucidate the connections of TFs, eRNA-related enhancers and downstream targets to better understand the oncogenicity of HCC.

In this study, we systematically explored the molecular biological mechanisms of HCC and constructed clinical prognostic models by integratively analysing the links among TFs, enhancers and downstream targets using bioinformatics to provide novel targets for HCC treatment.

## 2 Materials and methods

### 2.1 Data processing and sample materials

RNA-Seq and clinical information of 371 HCC tumours and 50 para-carcinoma tissues generated were downloaded from The Cancer Genome Atlas (TCGA, https://tcga-data.nci.nih.gov/) database, 110 normal tissues from the Genotype-Tissue Expression (GTEx) data portal (https://www.gtexportal.org/home/index.html) and the Gene Expression Omnibus (GEO, https://www.ncbi.nlm.nih.gov/gds) database. A total of 30 HCC tissues and paired adjacent non-tumor tissues were obtained by surgical resection from HCC patients. Two human HCC cell lines (HepG2, Huh7) and the immortal human liver cell line LO2 were obtained from the Shanghai Institute of Biochemistry and Cell Biology, Chinese Academy of Sciences, China. All of these patients have signed informed consents and this study was approved by the Ethics Committee of The First Affiliated Hospital of Ningbo University (KS20233004).

### 2.2 Identification of HCC-specific TFs and eRNA-related enhancers

The human Tissue-specific Enhancer Database (TiED, https://lcbb.swjtu.edu.cn/TiED) is a free, web-accessible database that provides much information about enhancers, eRNAs and interaction maps of enhancers, TFs and genes according to high-throughput sequencing data (Ernst and Kellis, 2010). In TiED, enhancers were identified based on four recognized features: H3K27ac, H3K4me1, H3K4me3, and DHS. ChIP-seq data were downloaded from NIH roadmap and ENCODE. Histone-binding regions were obtained by MACS (*p* < 0.0001) (Li et al., 2016). We extracted HCC-specific TF and eRNA-related enhancer information from TiED.

### 2.3 Identification of downstream targets of HCC-specific eRNA-related enhancers

EnhancerDB (http://lcbb.swjtu.edu.cn/EnhancerDB/) is a web database used to discover relationships in the context of various enhancers, allowing users to define tissue-specific enhancers by setting the threshold score of the tissue specificity of enhancers (Kang et al., 2019). We browsed and searched the HCC-related mRNAs, lncRNAs, and miRNAs regulated by the enhancers collected from TiED.

### 2.4 Construction of the TF-enhancer-target regulatory network

Differentially expressed TFs (DE-TFs) were screened from the TCGA database (*p* < 0.05). After the data processing was complete, the corresponding DE-TFs, eRNA-related enhancers, mRNAs and lncRNAs were used to construct the TF-enhancer-target network by Cytoscape version 3.8.0 software. The top 10 Hubba nodes were identified based on the cytoHubba plug-in with the degree algorithm (Su et al., 2022a).

### 2.5 Identification of coregulatory differentially expressed prognosis-related genes

The mRNAs coregulated by multiple enhancers and TFs were considered key genes. Differentially expressed genes (DEGs) were screened from the TCGA database (*p* < 0.05). We used Kaplan‒Meier plotter (http://kmplot.com/analysis/), a capable tool to assess the correlation between the expression of all genes and survival in 21 tumour types from several databases, including GEO and TCGA, to identify prognosis-related genes (Lanczky and Gyorffy, 2021). The protein expression of DAPK1 in HCC was compared using clinical proteomic tumor analysis consortium (CPTAC) samples in the University of ALabama at Birmingham CANcer data analysis Portal (UALCAN, https://ualcan.path.uab.edu/index.html) (Darshan et al., 2022).

### 2.6 Establishment and validation of the prognostic nomogram model

We selected some common clinical characteristics and the expression levels of the prognosis-related genes to construct a prognostic nomogram to predict the 1-, 3-, and 5-year OS rates of HCC patients using the R packages rms (version 6.2-0) and survival (version 3.2-10) in R software (R version 3.2.3). Moreover, the concordance index (C-index) was used to evaluate the discrimination of the nomogram, and calibration plots were used to show the association between the predicted and observed probabilities. Decision curve analysis (DCA) was built to evaluate the clinical net benefit (Chi et al., 2022a; Zhao et al., 2022b)

### 2.7 Functional analysis

KEGG enrichment analysis and Gene Ontology (GO) classification were performed for internal functional analysis using the R package clusterProfiler (version 3.14.3) (Chi et al., 2022b; Chi et al., 2022c). Methylation analysis was performed using the R package ggplot2 (version 3.3.3) in the TCGA cohort.

### 2.8 Immune infiltration analysis

We explored the associations between prognosis-related genes and the abundance of several infiltrating immune cells in HCC by the R package GSVA (version 1.34.0). The stromal, immune, estimate scores were calculated using the R package estimate (version 1.0.13) with the default parameters (Chi et al., 2022d; Peng et al., 2022). Tumor Immunization Single Cell Center (TISCH, http://tisch.comp-genomics.org/home/) is a single-cell RNA sequencing database about tumor microenvironment which was performed to detect the purity and immune infiltration of HCC (Sun et al., 2021). TISIDB (http://cis.hku.hk/TISIDB/index.php) is a web portal integrating multiple heterogeneous data types that was used for detecting HCC and immune system interactions (Ru et al., 2019). Pearson’s correlation analysis was conducted to determine the relationship between the expression of selected genes and indicators (*p* < 0.05).

### 2.9 Quantitative real-time polymerase chain reaction (qRT-PCR)

Cell and tissue RNA were extracted using TRIzol (Ambion, Carlsbad, United States) and reversed transcribed to cDNA with a GoScript Reverse Transcription (RT) System (Promega, Madison, United States). Subsequently, qRT-PCR detection was perfoermed with GoTaq qPCR Master Mix (Promega) whose conditions were as follows: 95°C for 5 min, followed by 40 cycles of 94°C for 15 s, 50°C for 30 s, and 72°C for 30 s. GAPDH mRNA was chosen to normalize and the primer sequences were as follows: DAPK1: forward, 5′-TTC​TGT​TGC​TAT​GAC​TAC​TTT​GCT​G-3′, reverse, 5′- AGG​ATG​TAT​CCT​TGT​CAT​ATC​CAA​A-3’; GAPDH: forward, 5′-ACC​CAC​TCC​TCC​ACC​TTT​GAC-3′, reverse, 5′-TGT​TGC​TGT​AGC​CAA​ATT​CGT​T-3’. Δ*C*t method was used to quantify (Δ*C*t = *C*t_DAPK1_- *C*t_GAPDH_). A higher Δ*C*t value means a lower DAPK1 level.

### 2.10 Statistical analysis

All analyses in the study were performed *via* R software (R version 3.2.3) or Graphpad version 8.02 and its support packages as mentioned before. Cytoscape version 3.8.0 software and its plug-in were used to build the network. *p* < 0.05 was considered significant.

## 3 Results

### 3.1 Identification of HCC-specific DE-TFs, eRNA-related enhancers and downstream targets

**FIGURE 1:**
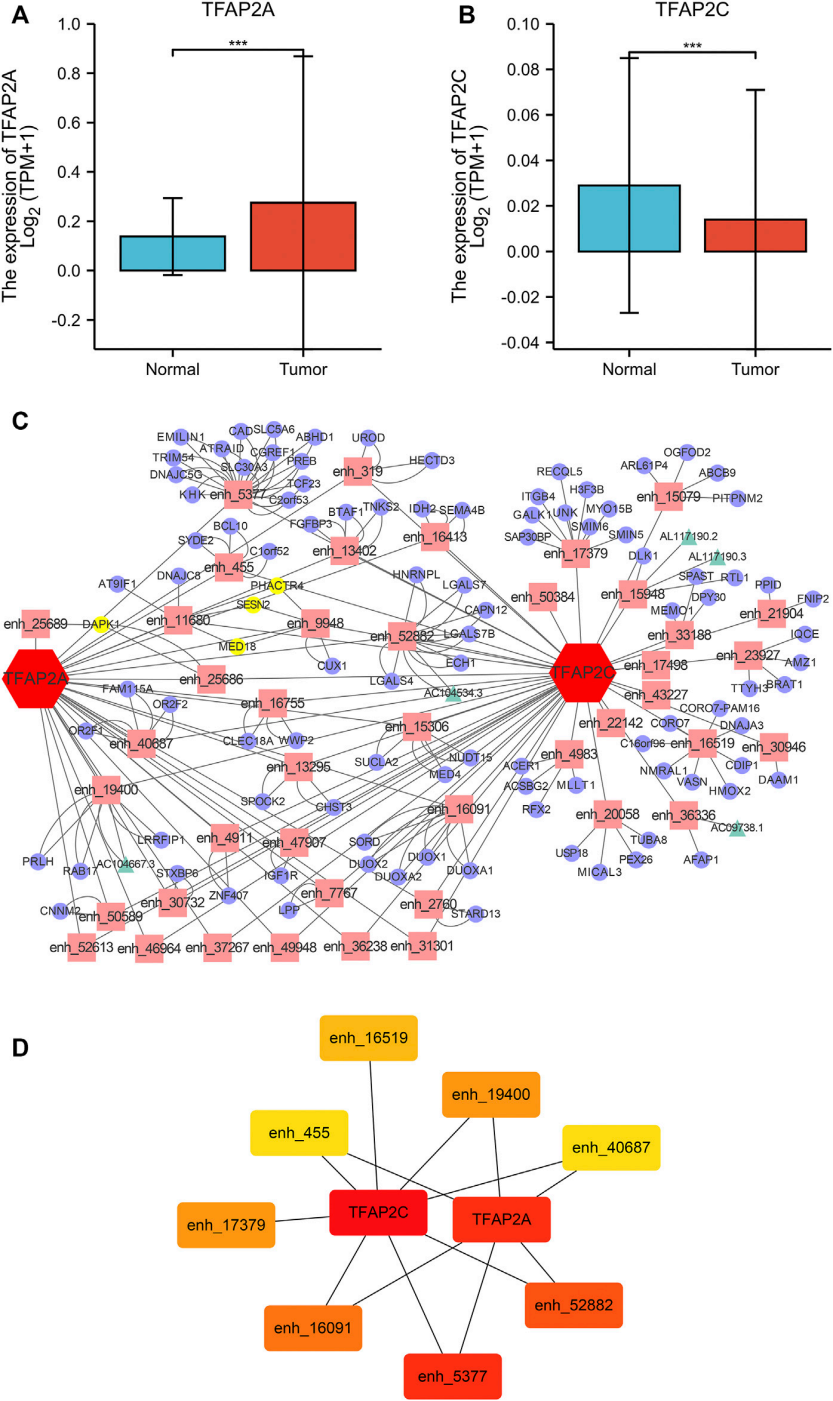
TF-Enhancer-Target Regulatory Network in HCC. **(A)** TFAP2A was significantly overexpression in HCC tissues. **(B)** TFAP2C was significantly downregulated in HCC tissues. **(C)** The interaction network map of TFs, eRNA associated enhancers, mRNAs and lncRNAs (TFs, red hexagon; enhancers, pink rectangle; mRNAs, purple cycle; lncRNAs, green triangle; co-regulatory targets, yellow node). **(D)** The heatmap of the hub targets in the regulatory network (*p < 0.05, **p < 0.01,***p < 0.001).

Twenty-seven HCC-specific TFs were listed in the TiED database, including ARID3A, BACH1, BATF, BCL11A, CEBPB, CTCFL, EGR1, ESR1, FOS, FOXA1, FOXA2, GATA1, GATA2, GATA3, HNF4A, HNF4G, IRF4, MAFF, MAFK, MTA3, MYBL2, MYC, PAX5, RUNX3, TEAD4, TFAP2A, and TFAP2C. We investigated the expression levels of these TFs, and only TFAP2A and TFAP2C were differentially expressed in the TCGA and GTEx HCC cohorts, as displayed in Figure 1 (Figures 1A, B). Then, the enhancers regulated by TFAP2A and TFAP2C in HCC tissue were identified. Our results showed that TFAP2A regulated 125 enhancers and that TFAP2C regulated 241 enhancers, as shown in Supplementary Table S1. We filtered and chose enhancers with the potential for transcription into eRNAs for further research. The mRNAs and lncRNAs regulated by these enhancers in the HepG2 cell line were output from EnhancerDB. We built the TF-enhancer-target regulatory network *via* Cytoscape, as displayed in Figure 1C. Our network contains 152 nodes (43 enhancers, 5 lncRNAs, and 104 mRNAs) and 238 edges. We used the cytoHubba plug-in in Cytoscape and identified 8 key hub enhancers (Figure 1D).

### 3.2 Identification of coregulatory differentially expressed prognosis-related genes

**FIGURE 2:**
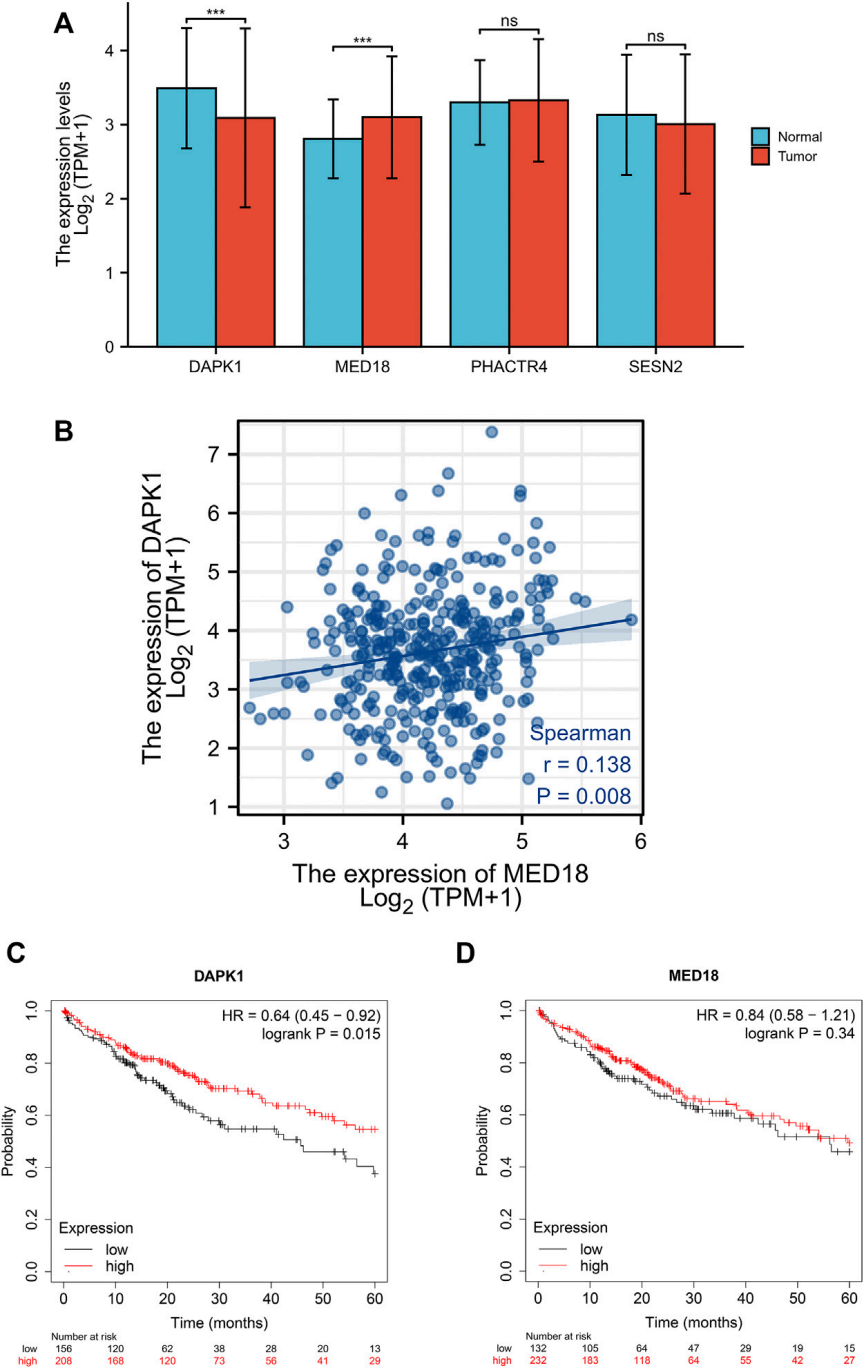
Identification of co-regulatory differentially expressed prognosis-related genes. **(A)** The expression level of co-regulatory genes between TCGA and GTEx cohort. **(B)** The expression association between DAPK1 and MED18. **(C)** Kaplan-Meier analysis of DAPK1 expression levels and overall survival. **(D)** Kaplan-Meier analysis of MED18 expression levels and overall survival (*p < 0.05, **p < 0.01, ***p < 0.001).

There were 4 mRNAs coregulated by multiple enhancers and TFs, including DAPK1, MED18, PHACTR4, and SESN2. We first detected the expression levels of these genes in the TCGA and GTEx databases. We found that DAPK1 was significantly downregulated and MED18 was overexpressed in HCC samples (Figure 2A). Coexpression analysis showed that DAPK1 and MED18 had highly correlated expression (Figure 2B). Then, we used Kaplan‒Meier plotter to analyse the prognostic characteristics of these genes. The results suggested that lower DAPK1 expression was associated with poor overall survival (OS) in HCC patients (Figure 2C). The expression of MED18 had no significant association with prognosis (Figure 2D). We further analysed the correlation between DAPK1 expression and clinicopathological factors in the TCGA cohort, as shown in Table 1. We found that age and gender were remarkably correlated with the expression level of DAPK1. Moreover, we further explored the diagnostic value of DAPK1 and built the ROC curve, which suggested that DAPK1 can be a potential screening biomarker of HCC (AUC = 0.630, Supplementary Figure S1).

**TABLE 1:** Correlation between DAPK1 expression levels in HCC samples and clinicopathological parameters.

Characteristic	Low expression of DAPK1	High expression of DAPK1	P
(%)	187 (50.0%)	187 (50.0%)	
T stage			0.543
T1	94 (25.3%)	89 (24%)	
T2	51 (13.7%)	44 (11.9%)	
T3	36 (9.7%)	44 (11.9%)	
T4	5 (1.3%)	8 (2.2%)	
N stage			0.622
N0	126 (48.8%)	128 (49.6%)	
N1	1 (0.4%)	3 (1.2%)	
M stage			0.623
M0	137 (50.4%)	131 (48.2%)	
M1	3 (1.1%)	1 (0.4%)	
Pathologic stage			0.205
Stage I	88 (25.1%)	85 (24.3%)	
Stage II	48 (13.7%)	39 (11.1%)	
Stage III	36 (10.3%)	49 (14%)	
Stage IV	4 (1.1%)	1 (0.3%)	
Tumor status			0.115
Tumor free	108 (30.4%)	94 (26.5%)	
With tumor	68 (19.2%)	85 (23.9%)	
Gender			0.008
Female	48 (12.8%)	73 (19.5%)	
Male	139 (37.2%)	114 (30.5%)	
Race			0.343
Asian	83 (22.9%)	77 (21.3%)	
Black or African American	10 (2.8%)	7 (1.9%)	
White	84 (23.2%)	101 (27.9%)	
Age			0.001
≤60	72 (19.3%)	105 (28.2%)	
>60	114 (30.6%)	82 (22%)	
BMI			0.864
≤25	88 (26.1%)	89 (26.4%)	
>25	82 (24.3%)	78 (23.1%)	
Histologic grade			0.303
G1	29 (7.9%)	26 (7%)	
G2	96 (26%)	82 (22.2%)	
G3	54 (14.6%)	70 (19%)	
G4	7 (1.9%)	5 (1.4%)	
Adjacent hepatic tissue inflammation			0.469
None	54 (22.8%)	64 (27%)	
Mild	47 (19.8%)	54 (22.8%)	
Severe	11 (4.6%)	7 (3%)	
AFP (ng/mL)			0.206
≤400	114 (40.7%)	101 (36.1%)	
>400	28 (10%)	37 (13.2%)	
Albumin (g/dL)			0.833
<3.5	36 (12%)	33 (11%)	
≥3.5	115 (38.3%)	116 (38.7%)	
Child-Pugh grade			0.807
A	120 (49.8%)	99 (41.1%)	
B	12 (5%)	9 (3.7%)	
C	0 (0%)	1 (0.4%)	
Prothrombin time			0.949
≤4	103 (34.7%)	105 (35.4%)	
>4	43 (14.5%)	46 (15.5%)	
Fibrosis ishak score			0.763
0	38 (17.7%)	37 (17.2%)	
1/2	17 (7.9%)	14 (6.5%)	
3/4	13 (6%)	15 (7%)	
5/6	46 (21.4%)	35 (16.3%)	
Vascular invasion			0.849
No	104 (32.7%)	104 (32.7%)	
Yes	57 (17.9%)	53 (16.7%)	

### 3.3 Construction and validation of the prognostic nomogram model

**FIGURE 3:**
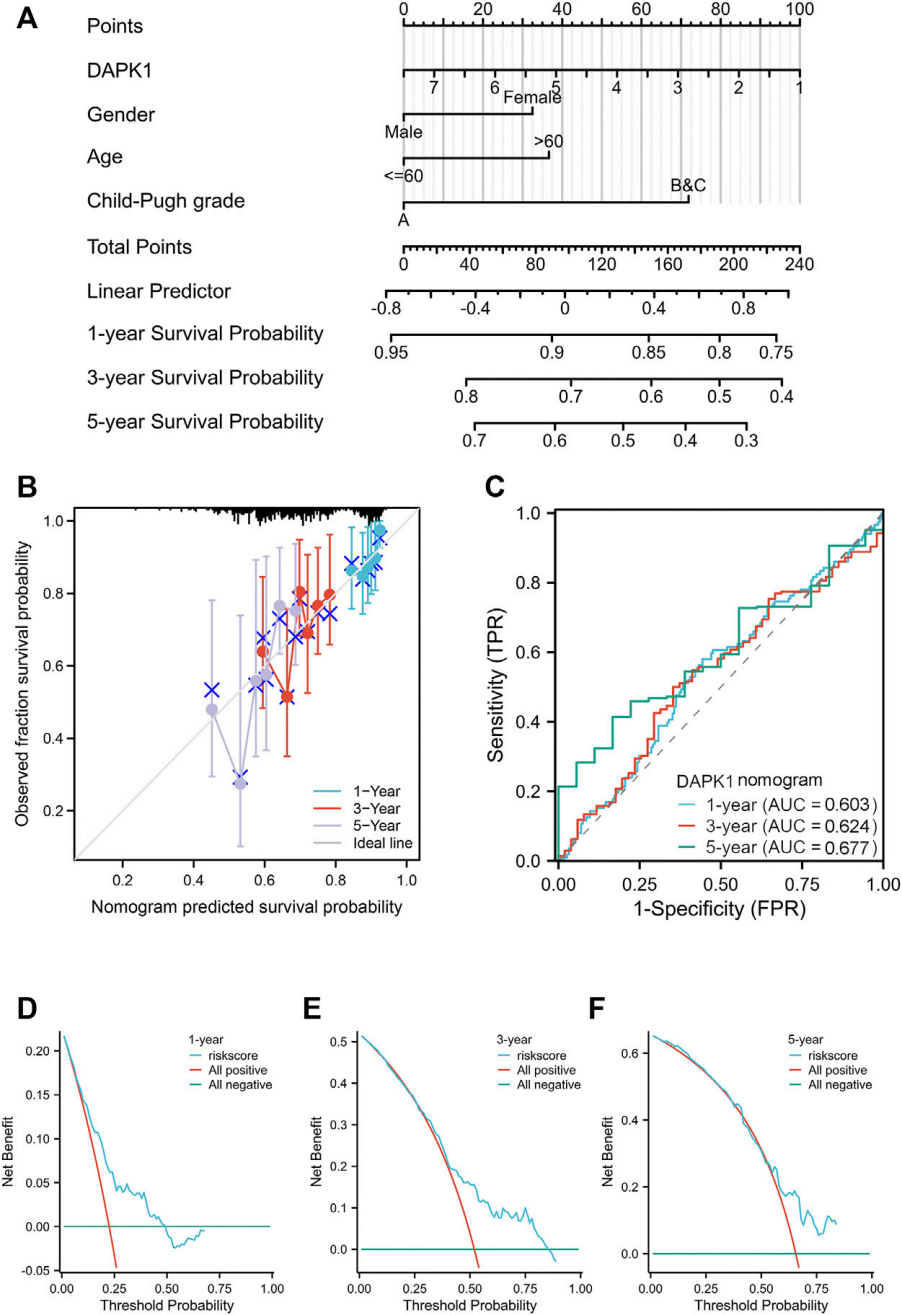
The overall survival nomogram model and calibration plots. **(A)** The overall survival nomogram model to predict the 1, 3, and 5 year OS of HCC patients. **(B)** The calibration plots of the nomogram model. **(C)** The 1, 3, and 5 year ROC curves of the nomogram. **(D–F)** The 1, 3, and 5 year DCA curves of the nomogram.

We built a nomogram model to predict the 1-, 3-, and 5-year OS of HCC patients (Figure 3A). The C-index for OS was 0.617 (95% CI: 0.586-0.649). Age and gender were included in our model according to the table of clinicopathological factors. Liver cirrhosis and liver function are important factors for HCC prognosis and quality of life (Kudo et al., 2020). Hence, we used Child‒Pugh scores, a versatile and simple tool that contains several clinical indicators, to evaluate liver function (Garcia-Tsao et al., 2019). PH assumptions and VIF assumptions have performed before the nomogram construction (Supplementary Tables S1–S3) and samples were combined according to the number of subjects. The calibration curves showed a satisfying consensus between the OS prediction by the nomogram and actual observation, as shown in Figure 3B for validation. The 1-, 3-, and 5-year ROC curves showed the nomogram has an ideal AUC in Figure 3C. Meanwhile, the DCA curves also indicated that this model was a practical model for clinical application (Figures 3D–F). In addition, we assessed the single clinicopathological factors and established Kaplan‒Meier curves, which should these factors could not be an independent prognostic factor in Supplementary Figure S2.

### 3.4 Functional analysis of the regulatory network

We selected protein-coding genes to understand the roles of these proteins in HCC carcinogenesis. KEGG analysis and GO analysis showed that our regulatory network mainly regulated the function of hydrogen peroxide, antibiotic metabolic process, and cofactor biosynthetic process, as shown in Supplementary Figure S3.

### 3.5 Exploration of the mechanism of DAPK1 in the prognosis of HCC

**FIGURE 4:**
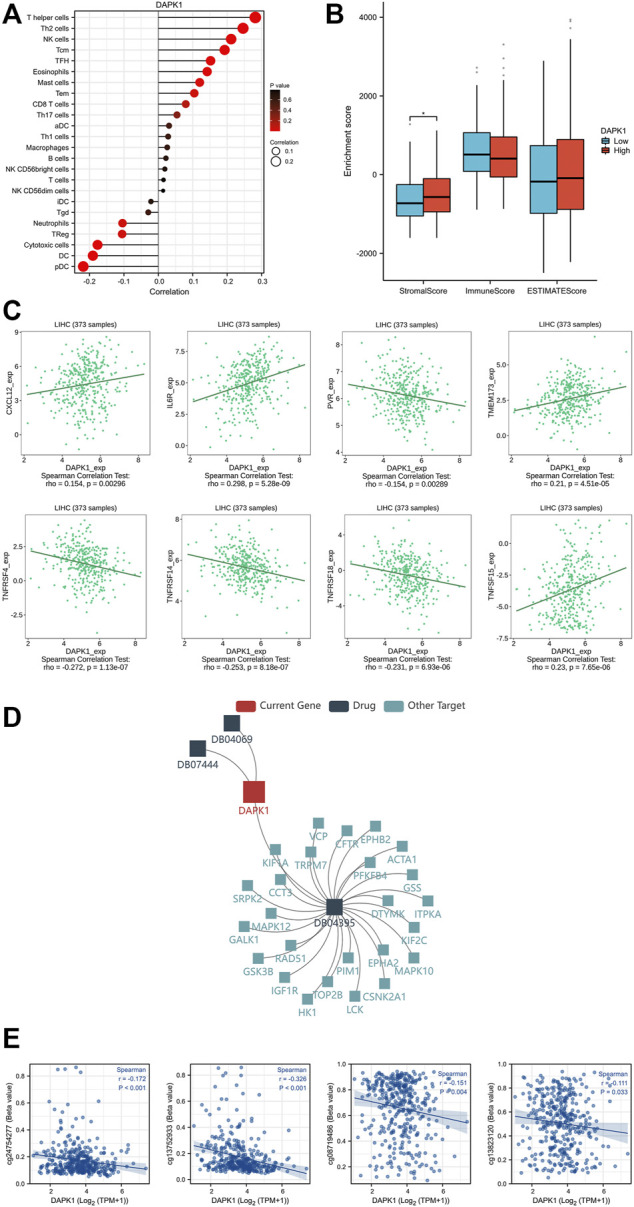
Immune cell infiltration levels and DNA methylation of DAPK1. **(A)** The lollipop diagram of the relationship between the expression of DAPK1 and immune cell infiltration levels. **(B)** The stromal score, immune score, estimate score of different expression level of DAPK1 in HCC. **(C)** The association between the expression of DAPK1 and immunostimulators. **(D)** The association between the expression of DAPK1 and target drugs. **(E)** The DNA methylation level of DAPK1.

We further explored the relationships between the prognostic significance of DAPK1 and its biological mechanisms. It has been demonstrated that HCC is always accompanied by an imbalance in immune cell infiltration (Haggitt et al., 1985). Accordingly, we first detected the infiltration landscape of immune cells in HCC and found quantitative differences in several immune cells, such as macrophages, mast cells, NK cells, T helper cells, Th1 cells, Th17 cells, Th2 cells, and Tregs, as shown in lollipop plots (Figure 4A) and Supplementary Table S4. The stromal score revealed that the expression of DAPK1 was negatively associated with stromal cells in HCC (Figure 4B). The association between the expression of DAPK1 and immunostimulators was shown in Figure 4C. Moreover, the expression of DAPK1 correlated with several targeting drugs (DB04069: 5,6-Dihydro-Benzo [H]Cinnolin-3-Ylamine, DB04395: Phosphoaminophosphonic Acid-Adenylate Ester, DB07444: 6-(3-AMINOPROPYL)-4,9-DIMETHYLPYRROLO [3,4-C]CARBAZOLE-1,3(2H, 6H)-DIONE), as shown in Figure 4D, which implied considerable promise for immunotherapy in HCC.

DNA methylation is one of the most predominant forms of epigenetic modifications that modulates downstream gene regulation (Yousefi et al., 2022). We observed multiple methylated sites in the promoter and enhancer regions of DAPK1 (Figure 4E), which is worthy of further validation in future studies.

### 3.6 Analysis of the HCC immune microenvironment of DAPK1

**FIGURE 5:**
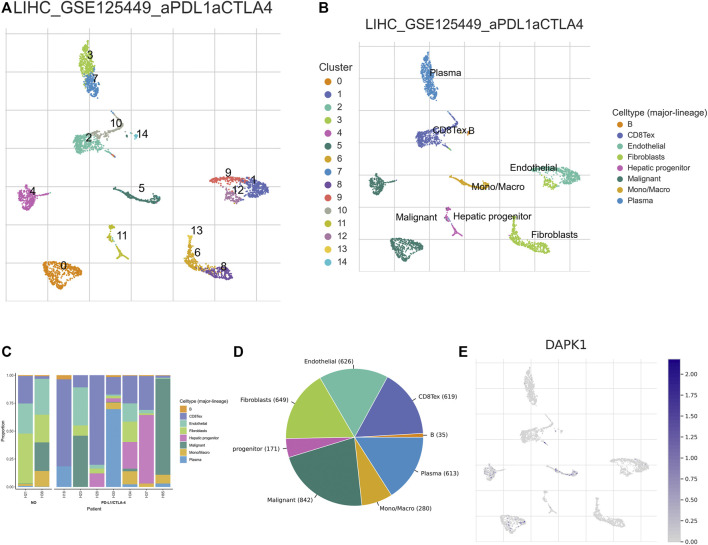
Analysis of the expression of DAPK1 in HCC immune microenvironment. **(A–D)** Annotation of all cell types in LIHC_GSE125449 and the percentage of each cell type. **(E)** The percents of the expression of DAPK1 in HCC.

The expression of DAPK1 in the microenvironment of HCC was analyzed. There were 14 cell populations and 8 immune cell types in the LIHC_GSE125449 dataset (Figures 5A, B), which displayed the distribution and number of various cell types (Figures 5C, D). DAPK1 in immune cells was mainly expressed in malignant HCC cells and macrophages but barely expression in the fibroblast in the immune microenvironment (Figure 5E).

### 3.7 Validation of the differential expression of DAPK1

**FIGURE 6:**
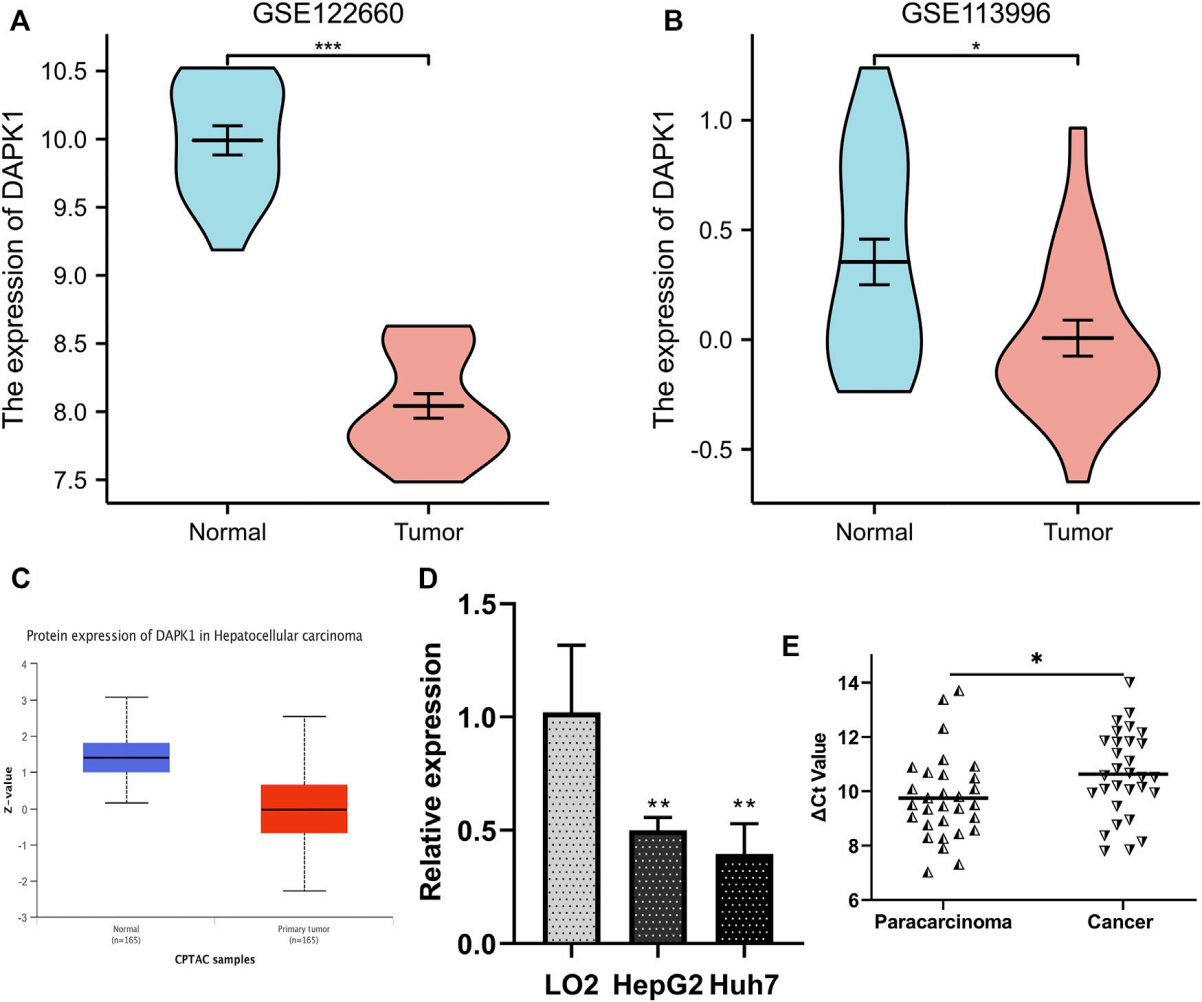
Validation of the differentially expression level of DAPK1 in GEO database. **(A)** DAPK1 was downregulated in HCC cells from GSE122660. **(B)** DAPK1 was downregulated in HCC tissues from GSE113996. **(C)** The protein level of DAPK1 was downregulated in UALCAN cohort. **(D)** DAPK1 was downregulated in HepG2, Huh7 cell compared to the LO2. **(E)** DAPK1 was downregulated in HCC tissues (*p < 0.05, **p < 0.01, ***p < 0.001).

We compared the expression level of DAPK1 in the cell line and tissue line from the GEO database for validation. The mRNA sequencing profiles of the HCC cell line and normal control were obtained from GEO (accession numbers: GSE122660, submission date: 17 November 2018; last update date: 19 December 2017) (Stephanie et al., 2018). The mRNA sequencing profiles of HCC tissue and normal control tissue were obtained from GSE113996 (submission date: 03 May 2018; last update date: 04 May 2021, https://ncbi.nlm.nih.gov/geo/query/acc.cgi?acc=GSE113996). The results revealed that DAPK1 expression was prominently lower in the HCC samples (Figures 6A, B), which was consistent with that in previous studies. Likewise, the protein expression of DAPK1 was also lower in HCC compared using CPTAC samples in UALCAN database (Figure 6C). Moreover, we performed qRT-PCR for further validation and our results showed that DAPK1 was downregulated in the HCC cell line and tissue line (Figures 6D, E). All of the evidence supported our results.

## 4 Discussion

Recently, the roles of enhancers in malignant tumours have been increasingly focused on by researchers. It has been confirmed that enhancers regulate tumour proliferation, cell migration, angiogenesis, and apoptosis and are further involved in tumour resistance, occurrence, and development. eRNA is a novel kind of lncRNA that effectively contributes to enhancer activity and function (Morton et al., 2019). Moreover, eRNA-associated enhancers always have a higher ability to bind transcriptional coactivators and marks of active chromatin than non-transcribed enhancers (Marchese et al., 2017). Thus, the intrinsic connections of eRNA-associated enhancers became our focus.

In this study, we identified differentially expressed TFs, eRNA-associated enhancers, and downstream targets and built a novel regulatory network. We next ascertained the coregulatory differentially expressed prognosis-related genes and constructed a nomogram model to predict the long-term survival time. Then, we explored the potential functions and the regulatory mechanisms of methylation modification and immune cell infiltration levels. Finally, we verified the differential expression of DAPK1 in cell lines and tissue lines from the GEO and UALCAN database. qRT-PCR was used for further validation in the cell and tissue line.

Death-associated protein kinase 1 (DAPK1) is a positive mediator of interferon-gamma-induced programmed cell death and is a tumour suppressor candidate that inhibits tumour immune evasion in gastric cancer (Guo et al., 2022). Our study revealed that DAPK1 is a key suppressor prognostic gene in HCC, and the data in the GEO database also supported our results. We further designed and validated a nomogram model to predict OS with high confidence and value for clinical application. As we all known, “Child-Pugh grade” is a practical parameter to assess function of liver, which is derived from multiple clinical indicators such as TBil (total bilirubin), albumin (alb), plasma prothrombin coagulative time (PT), ascites and the level of hepatic encephalopathy. However, it is difficult to find a cohort completely containing all of the parameters in our nomogram especially “Child-Pugh grade” or its clinical indicators in GEO database and International Cancer Genome Consortium (ICGC) database. Meanwhile, it is burdensome to collect and finish RNA-Seq for enough samples at once. We wish this model can be further tested and validated by large cohort.

Targeted therapies and immune therapy approaches targeting mutation-associated neoantigens are promising strategies for HCC, especially in non-surgical treatment (Roth et al., 2022). Immunotherapy can greatly activate the autoimmunity of HCC patients but the therapeutic performance in clinical trials remain unsatisfactory till now (Chen et al., 2022). For example, the objective response rate of HCC was still low in the patient received combination therapy of PD-1/PD-L1 inhibitors and targeted drugs (Su et al., 2022b). In this work, we analyzed the tumor microenvironment and found that the prognosis-related gene DAPK1 was correlated with several immune cells, immunostimulators and targeting drugs. However, experimental and clinical validation of this conclusion is needed.

In conclusion, we established a significant TF-enhancer-target regulatory network and identified downregulated DAPK1 as an important prognostic and diagnostic gene in HCC. The potential biological function and mechanism were annotated using bioinformatics tools.

## Data Availability

The datasets presented in this study can be found in online repositories. The names of the repository/repositories and accession number(s) can be found in the article/Supplementary Material.
