# Supplementary material for: Identification of novel prognostic biomarkers in the TF-enhancer-target regulatory network in hepatocellular carcinoma and immune infiltration analysis
Source: Front Genet. 2023 Mar 29;14:1158341. doi: 10.3389/fgene.2023.1158341 (PMC10090374; doi:10.3389/fgene.2023.1158341)
Supplement: Supplementary file 1 [file DataSheet1.DOCX]

Supplementary Material

Identification of Novel Prognostic Biomarkers in the TF-Enhancer-Target Regulatory Network in Hepatocellular Carcinoma and Immune Infiltration Analysis

Jianing Yan, Guoliang Ye, Yongfu Shao*, Hanxuan Zhou

*** Correspondence:** Yongfu Shao: fyshaoyongfu@nbu.edu.cn

# Supplementary Figures and Tables

##
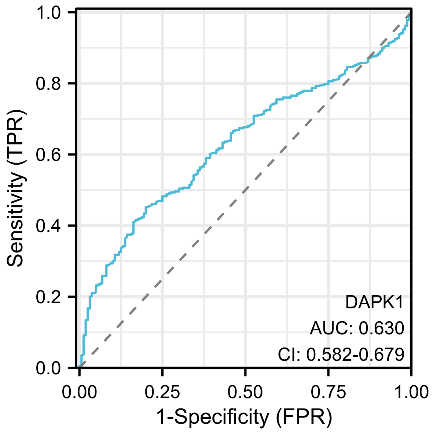
Supplementary Figures

**Supplementary Figure 1.** Diagnostic value analysis of the DAPK1.


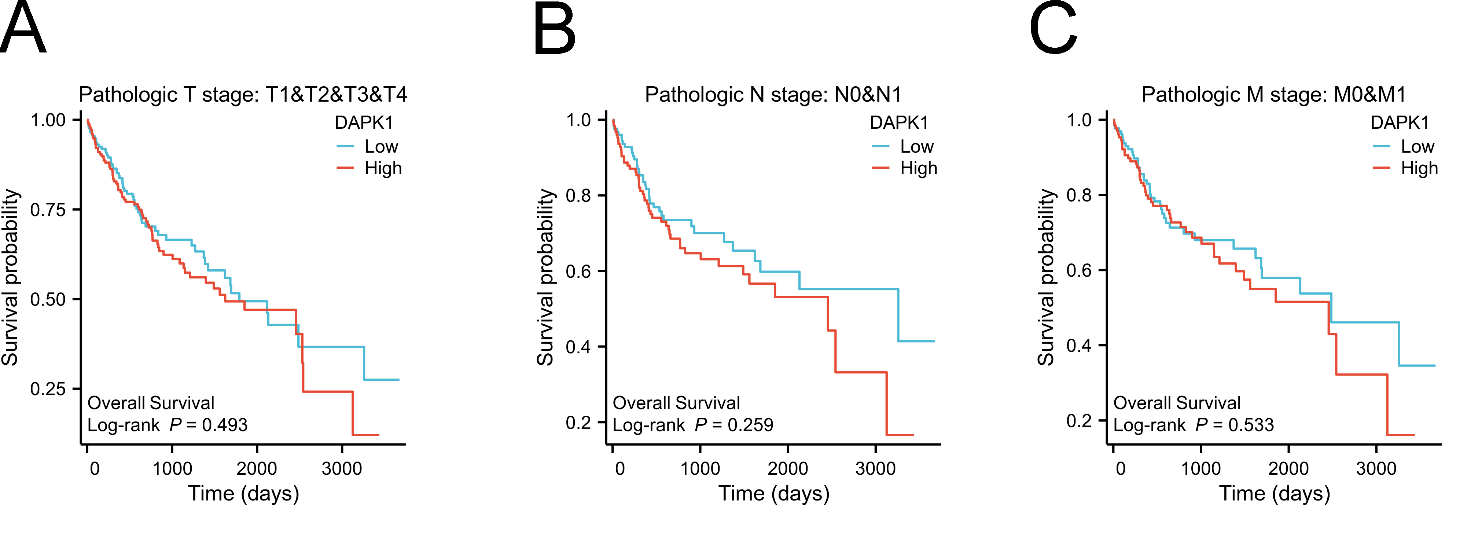


**Supplementary Figure 2.** Prognostic value analysis of the clinicopathological factors.


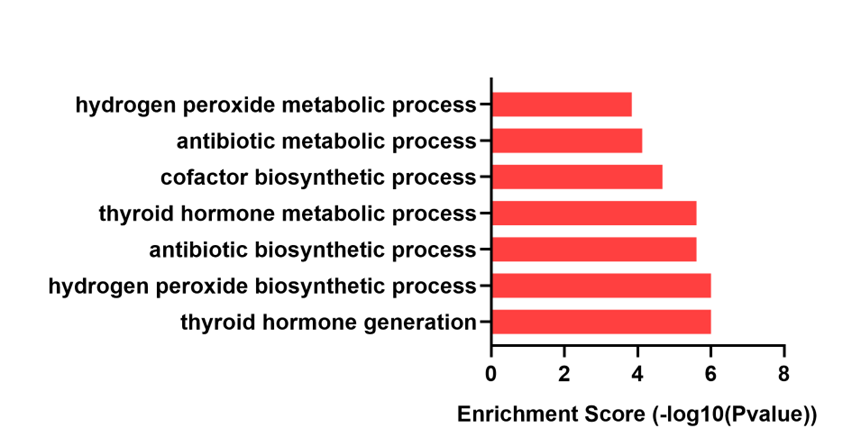


**Supplementary Figure 3.** Functional analysis of the regulatory network

## Supplementary Tables

| **Supplementary Table 1** TFs and regulated enhancers | | | |
| --- | --- | --- | --- |
| TFs | Location | Enhancer | eRNA |
| TFAP2A | chr1:935357-935970 | enh_12 | 2D-eRNA |
| TFAP2A | chr1:234859860-234860486 | enh_5377 | 2D-eRNA |
| TFAP2A | chr10:74020291-74020639 | enh_7159 | 2D-eRNA |
| TFAP2A | chr11:76482022-76482301 | enh_9945 | 2D-eRNA |
| TFAP2A | chr16:87823505-87824169 | enh_19885 | 2D-eRNA |
| TFAP2A | chr19:2059664-2059999 | enh_23267 | 2D-eRNA |
| TFAP2A | chr19:13273536-13274192 | enh_23505 | 2D-eRNA |
| TFAP2A | chr5:133841780-133842373 | enh_40692 | 2D-eRNA |
| TFAP2A | chr5:172141844-172142280 | enh_41592 | 2D-eRNA |
| TFAP2A | chr6:6857268-6857681 | enh_42161 | 2D-eRNA |
| TFAP2A | chr1:935357-935970 | enh_12 | 2D-eRNA |
| TFAP2A | chr1:234859860-234860486 | enh_5377 | 2D-eRNA |
| TFAP2A | chr10:74020291-74020639 | enh_7159 | 2D-eRNA |
| TFAP2A | chr11:76482022-76482301 | enh_9945 | 2D-eRNA |
| TFAP2A | chr16:87823505-87824169 | enh_19885 | 2D-eRNA |
| TFAP2A | chr19:2059664-2059999 | enh_23267 | 2D-eRNA |
| TFAP2A | chr19:13273536-13274192 | enh_23505 | 2D-eRNA |
| TFAP2A | chr5:133841780-133842373 | enh_40692 | 2D-eRNA |
| TFAP2A | chr5:172141844-172142280 | enh_41592 | 2D-eRNA |
| TFAP2A | chr6:6857268-6857681 | enh_42161 | 2D-eRNA |
| TFAP2A | chr1:12600066-12600677 | enh_386 | 1D-eRNA |
| TFAP2A | chr1:16499996-16500474 | enh_480 | 1D-eRNA |
| TFAP2A | chr1:95417138-95417434 | enh_2399 | 1D-eRNA |
| TFAP2A | chr1:110334836-110335137 | enh_2524 | 1D-eRNA |
| TFAP2A | chr1:202075404-202075870 | enh_4185 | 1D-eRNA |
| TFAP2A | chr1:202076390-202077004 | enh_4185 | 1D-eRNA |
| TFAP2A | chr1:223254480-223254811 | enh_4911 | 1D-eRNA |
| TFAP2A | chr10:99447050-99447719 | enh_7767 | 1D-eRNA |
| TFAP2A | chr11:76482022-76482301 | enh_9944 | 1D-eRNA |
| TFAP2A | chr12:6387813-6388373 | enh_11226 | 1D-eRNA |
| TFAP2A | chr12:125165964-125166681 | enh_13467 | 1D-eRNA |
| TFAP2A | chr12:132345295-132345769 | enh_13618 | 1D-eRNA |
| TFAP2A | chr14:35806091-35806306 | enh_15306 | 1D-eRNA |
| TFAP2A | chr14:105663587-105663874 | enh_16677 | 1D-eRNA |
| TFAP2A | chr15:41198241-41198568 | enh_17034 | 1D-eRNA |
| TFAP2A | chr15:41198580-41198893 | enh_17034 | 1D-eRNA |
| TFAP2A | chr16:85548569-85549036 | enh_19739 | 1D-eRNA |
| TFAP2A | chr17:62403831-62404284 | enh_21456 | 1D-eRNA |
| TFAP2A | chr19:49314251-49314479 | enh_24150 | 1D-eRNA |
| TFAP2A | chr2:43385418-43386067 | enh_25686 | 1D-eRNA |
| TFAP2A | chr20:30181128-30181877 | enh_30311 | 1D-eRNA |
| TFAP2A | chr20:30183490-30184245 | enh_30311 | 1D-eRNA |
| TFAP2A | chr20:36754878-36755319 | enh_30448 | 1D-eRNA |
| TFAP2A | chr20:48993509-48993864 | enh_30841 | 1D-eRNA |
| TFAP2A | chr3:194053926-194054563 | enh_36238 | 1D-eRNA |
| TFAP2A | chr4:74975253-74975553 | enh_37267 | 1D-eRNA |
| TFAP2A | chr7:100769740-100770317 | enh_46964 | 1D-eRNA |
| TFAP2A | chr8:17323871-17324174 | enh_48028 | 1D-eRNA |
| TFAP2A | chr8:144097350-144097695 | enh_50608 | 1D-eRNA |
| TFAP2A | chr9:93763470-93764115 | enh_51796 | 1D-eRNA |
| TFAP2A | chr9:124132406-124132996 | enh_52613 | 1D-eRNA |
| TFAP2A | chr9:132319690-132319986 | enh_52882 | 1D-eRNA |
| TFAP2A | chrX:41240922-41241318 | enh_53489 | 1D-eRNA |
| TFAP2A | chr1:10488072-10488441 | enh_319 | no-eRNA |
| TFAP2A | chr1:16126612-16127003 | enh_455 | no-eRNA |
| TFAP2A | chr1:16403442-16403724 | enh_466 | no-eRNA |
| TFAP2A | chr1:25900112-25900715 | enh_795 | no-eRNA |
| TFAP2A | chr1:95417138-95417434 | enh_2398 | no-eRNA |
| TFAP2A | chr1:110334836-110335137 | enh_2525 | no-eRNA |
| TFAP2A | chr1:116832906-116833300 | enh_2760 | no-eRNA |
| TFAP2A | chr1:173092169-173092810 | enh_3622 | no-eRNA |
| TFAP2A | chr1:181059054-181059590 | enh_3768 | no-eRNA |
| TFAP2A | chr1:202075404-202075870 | enh_4186 | no-eRNA |
| TFAP2A | chr1:202076390-202077004 | enh_4186 | no-eRNA |
| TFAP2A | chr1:207414811-207415096 | enh_4407 | no-eRNA |
| TFAP2A | chr1:211811970-211812501 | enh_4552 | no-eRNA |
| TFAP2A | chr1:234793938-234794185 | enh_5354 | no-eRNA |
| TFAP2A | chr1:234859860-234860486 | enh_5378 | no-eRNA |
| TFAP2A | chr10:93392864-93393441 | enh_7679 | no-eRNA |
| TFAP2A | chr10:112216458-112216900 | enh_7978 | no-eRNA |
| TFAP2A | chr10:116484063-116484410 | enh_8104 | no-eRNA |
| TFAP2A | chr11:47959577-47960203 | enh_9248 | no-eRNA |
| TFAP2A | chr11:75981533-75981820 | enh_9905 | no-eRNA |
| TFAP2A | chr11:76482022-76482301 | enh_9948 | no-eRNA |
| TFAP2A | chr12:46867481-46868072 | enh_11680 | no-eRNA |
| TFAP2A | chr12:76371654-76371957 | enh_12432 | no-eRNA |
| TFAP2A | chr12:116844355-116844697 | enh_13295 | no-eRNA |
| TFAP2A | chr12:122064338-122064948 | enh_13402 | no-eRNA |
| TFAP2A | chr12:125211750-125212162 | enh_13485 | no-eRNA |
| TFAP2A | chr12:132345295-132345769 | enh_13619 | no-eRNA |
| TFAP2A | chr13:95966735-95967082 | enh_14768 | no-eRNA |
| TFAP2A | chr13:110651092-110651469 | enh_14950 | no-eRNA |
| TFAP2A | chr14:35806091-35806306 | enh_15307 | no-eRNA |
| TFAP2A | chr14:75866987-75867228 | enh_16038 | no-eRNA |
| TFAP2A | chr14:77419759-77420425 | enh_16091 | no-eRNA |
| TFAP2A | chr14:99849445-99849791 | enh_16413 | no-eRNA |
| TFAP2A | chr15:31588925-31589224 | enh_16755 | no-eRNA |
| TFAP2A | chr15:41198241-41198568 | enh_17033 | no-eRNA |
| TFAP2A | chr15:70488753-70489158 | enh_17635 | no-eRNA |
| TFAP2A | chr15:70490108-70490669 | enh_17637 | no-eRNA |
| TFAP2A | chr15:70730216-70730846 | enh_17669 | no-eRNA |
| TFAP2A | chr15:77789565-77789845 | enh_17861 | no-eRNA |
| TFAP2A | chr16:73158296-73158905 | enh_19400 | no-eRNA |
| TFAP2A | chr16:85548569-85549036 | enh_19740 | no-eRNA |
| TFAP2A | chr16:87823505-87824169 | enh_19886 | no-eRNA |
| TFAP2A | chr17:40819487-40820122 | enh_20819 | no-eRNA |
| TFAP2A | chr17:41535230-41535532 | enh_20872 | no-eRNA |
| TFAP2A | chr17:49029411-49029688 | enh_21234 | no-eRNA |
| TFAP2A | chr19:13273536-13274192 | enh_23503 | no-eRNA |
| TFAP2A | chr19:18412022-18412612 | enh_23662 | no-eRNA |
| TFAP2A | chr19:19174206-19174573 | enh_23694 | no-eRNA |
| TFAP2A | chr2:11530022-11530552 | enh_24640 | no-eRNA |
| TFAP2A | chr2:28583298-28583571 | enh_25135 | no-eRNA |
| TFAP2A | chr2:42190743-42191179 | enh_25519 | no-eRNA |
| TFAP2A | chr2:42193622-42194288 | enh_25523 | no-eRNA |
| TFAP2A | chr2:43385418-43386067 | enh_25689 | no-eRNA |
| TFAP2A | chr20:30181128-30181877 | enh_30308 | no-eRNA |
| TFAP2A | chr20:30183490-30184245 | enh_30308 | no-eRNA |
| TFAP2A | chr20:30198529-30198897 | enh_30315 | no-eRNA |
| TFAP2A | chr20:46082142-46082409 | enh_30650 | no-eRNA |
| TFAP2A | chr20:48232238-48232623 | enh_30732 | no-eRNA |
| TFAP2A | chr21:16582447-16583082 | enh_31301 | no-eRNA |
| TFAP2A | chr21:34752960-34753190 | enh_31530 | no-eRNA |
| TFAP2A | chr21:40169533-40169741 | enh_31678 | no-eRNA |
| TFAP2A | chr3:49169754-49170330 | enh_33983 | no-eRNA |
| TFAP2A | chr3:193721240-193721638 | enh_36161 | no-eRNA |
| TFAP2A | chr3:194053926-194054563 | enh_36237 | no-eRNA |
| TFAP2A | chr3:194751449-194751974 | enh_36304 | no-eRNA |
| TFAP2A | chr4:38320649-38321179 | enh_36986 | no-eRNA |
| TFAP2A | chr4:74975253-74975553 | enh_37264 | no-eRNA |
| TFAP2A | chr4:78077671-78077974 | enh_37308 | no-eRNA |
| TFAP2A | chr5:33148415-33148624 | enh_38799 | no-eRNA |
| TFAP2A | chr5:133802194-133802787 | enh_40687 | no-eRNA |
| TFAP2A | chr5:168027430-168027833 | enh_41486 | no-eRNA |
| TFAP2A | chr5:172141844-172142280 | enh_41591 | no-eRNA |
| TFAP2A | chr5:173221276-173221443 | enh_41729 | no-eRNA |
| TFAP2A | chr5:176882419-176882695 | enh_41807 | no-eRNA |
| TFAP2A | chr6:16965079-16965305 | enh_42520 | no-eRNA |
| TFAP2A | chr6:33807882-33808545 | enh_42939 | no-eRNA |
| TFAP2A | chr6:52783057-52783501 | enh_43481 | no-eRNA |
| TFAP2A | chr7:27702642-27702856 | enh_46028 | no-eRNA |
| TFAP2A | chr7:155711745-155712350 | enh_47778 | no-eRNA |
| TFAP2A | chr8:8458386-8458669 | enh_47907 | no-eRNA |
| TFAP2A | chr8:40032230-40032636 | enh_48581 | no-eRNA |
| TFAP2A | chr8:109152143-109152497 | enh_49593 | no-eRNA |
| TFAP2A | chr8:126464267-126464654 | enh_49948 | no-eRNA |
| TFAP2A | chr8:126559358-126559693 | enh_49976 | no-eRNA |
| TFAP2A | chr8:126568644-126568911 | enh_49979 | no-eRNA |
| TFAP2A | chr8:126656706-126657003 | enh_50002 | no-eRNA |
| TFAP2A | chr8:143807806-143808423 | enh_50589 | no-eRNA |
| TFAP2C | chr1:935350-935602 | enh_12 | 2D-eRNA |
| TFAP2C | chr1:234659337-234659611 | enh_5294 | 2D-eRNA |
| TFAP2C | chr1:234859848-234860256 | enh_5377 | 2D-eRNA |
| TFAP2C | chr10:74020278-74020666 | enh_7159 | 2D-eRNA |
| TFAP2C | chr11:61740337-61740912 | enh_9395 | 2D-eRNA |
| TFAP2C | chr11:65259323-65259506 | enh_9541 | 2D-eRNA |
| TFAP2C | chr11:76481999-76482325 | enh_9945 | 2D-eRNA |
| TFAP2C | chr14:75760730-75760941 | enh_16015 | 2D-eRNA |
| TFAP2C | chr14:90849464-90850067 | enh_16223 | 2D-eRNA |
| TFAP2C | chr16:87823502-87823928 | enh_19885 | 2D-eRNA |
| TFAP2C | chr1:935350-935602 | enh_12 | 2D-eRNA |
| TFAP2C | chr1:234659337-234659611 | enh_5294 | 2D-eRNA |
| TFAP2C | chr1:234859848-234860256 | enh_5377 | 2D-eRNA |
| TFAP2C | chr10:74020278-74020666 | enh_7159 | 2D-eRNA |
| TFAP2C | chr11:61740337-61740912 | enh_9395 | 2D-eRNA |
| TFAP2C | chr11:65259323-65259506 | enh_9541 | 2D-eRNA |
| TFAP2C | chr11:76481999-76482325 | enh_9945 | 2D-eRNA |
| TFAP2C | chr14:75760730-75760941 | enh_16015 | 2D-eRNA |
| TFAP2C | chr14:90849464-90850067 | enh_16223 | 2D-eRNA |
| TFAP2C | chr16:87823502-87823928 | enh_19885 | 2D-eRNA |
| TFAP2C | chr19:2059650-2060270 | enh_23267 | 2D-eRNA |
| TFAP2C | chr19:13273489-13274169 | enh_23505 | 2D-eRNA |
| TFAP2C | chr3:194754092-194754608 | enh_36305 | 2D-eRNA |
| TFAP2C | chr5:133841724-133842352 | enh_40692 | 2D-eRNA |
| TFAP2C | chr5:172141775-172142323 | enh_41592 | 2D-eRNA |
| TFAP2C | chr6:6857250-6857719 | enh_42161 | 2D-eRNA |
| TFAP2C | chr6:43774108-43774366 | enh_43227 | 2D-eRNA |
| TFAP2C | chr8:61825354-61825640 | enh_48916 | 2D-eRNA |
| TFAP2C | chr1:8264618-8264844 | enh_173 | 1D-eRNA |
| TFAP2C | chr1:12221905-12222444 | enh_376 | 1D-eRNA |
| TFAP2C | chr1:12600006-12600658 | enh_386 | 1D-eRNA |
| TFAP2C | chr1:16499984-16500471 | enh_480 | 1D-eRNA |
| TFAP2C | chr1:56278095-56278339 | enh_1628 | 1D-eRNA |
| TFAP2C | chr1:95417136-95417445 | enh_2399 | 1D-eRNA |
| TFAP2C | chr1:110334803-110335456 | enh_2524 | 1D-eRNA |
| TFAP2C | chr1:202075385-202075885 | enh_4185 | 1D-eRNA |
| TFAP2C | chr1:202076371-202076691 | enh_4185 | 1D-eRNA |
| TFAP2C | chr1:202077431-202077775 | enh_4185 | 1D-eRNA |
| TFAP2C | chr1:223254464-223254856 | enh_4911 | 1D-eRNA |
| TFAP2C | chr1:234659337-234659611 | enh_5293 | 1D-eRNA |
| TFAP2C | chr1:235123189-235123451 | enh_5463 | 1D-eRNA |
| TFAP2C | chr10:99447036-99447704 | enh_7767 | 1D-eRNA |
| TFAP2C | chr11:18405670-18406232 | enh_8844 | 1D-eRNA |
| TFAP2C | chr11:47211116-47211268 | enh_9222 | 1D-eRNA |
| TFAP2C | chr11:76481999-76482325 | enh_9944 | 1D-eRNA |
| TFAP2C | chr12:6387800-6388362 | enh_11226 | 1D-eRNA |
| TFAP2C | chr12:125165964-125166595 | enh_13467 | 1D-eRNA |
| TFAP2C | chr12:132345256-132345788 | enh_13618 | 1D-eRNA |
| TFAP2C | chr14:35806010-35806347 | enh_15306 | 1D-eRNA |
| TFAP2C | chr14:103747838-103748087 | enh_16580 | 1D-eRNA |
| TFAP2C | chr14:105663579-105663898 | enh_16677 | 1D-eRNA |
| TFAP2C | chr15:41198213-41198878 | enh_17034 | 1D-eRNA |
| TFAP2C | chr15:67342080-67342360 | enh_17498 | 1D-eRNA |
| TFAP2C | chr16:29302211-29302506 | enh_18824 | 1D-eRNA |
| TFAP2C | chr16:85548382-85549087 | enh_19739 | 1D-eRNA |
| TFAP2C | chr17:62403842-62404358 | enh_21456 | 1D-eRNA |
| TFAP2C | chr17:76310003-76310719 | enh_21904 | 1D-eRNA |
| TFAP2C | chr19:49314241-49314496 | enh_24150 | 1D-eRNA |
| TFAP2C | chr2:43385411-43386052 | enh_25686 | 1D-eRNA |
| TFAP2C | chr20:30181109-30181903 | enh_30311 | 1D-eRNA |
| TFAP2C | chr20:30183420-30183966 | enh_30311 | 1D-eRNA |
| TFAP2C | chr20:36754851-36755386 | enh_30448 | 1D-eRNA |
| TFAP2C | chr20:48909033-48909758 | enh_30809 | 1D-eRNA |
| TFAP2C | chr20:48993495-48993891 | enh_30841 | 1D-eRNA |
| TFAP2C | chr20:52238238-52238887 | enh_30946 | 1D-eRNA |
| TFAP2C | chr3:170074585-170074894 | enh_35675 | 1D-eRNA |
| TFAP2C | chr3:194053928-194054762 | enh_36238 | 1D-eRNA |
| TFAP2C | chr3:195862843-195863356 | enh_36336 | 1D-eRNA |
| TFAP2C | chr4:24212086-24212330 | enh_36785 | 1D-eRNA |
| TFAP2C | chr4:74975231-74975567 | enh_37267 | 1D-eRNA |
| TFAP2C | chr5:139022996-139023287 | enh_40824 | 1D-eRNA |
| TFAP2C | chr5:173221147-173221523 | enh_41728 | 1D-eRNA |
| TFAP2C | chr6:37104995-37105208 | enh_43060 | 1D-eRNA |
| TFAP2C | chr6:37105247-37105467 | enh_43060 | 1D-eRNA |
| TFAP2C | chr6:37483660-37484375 | enh_43074 | 1D-eRNA |
| TFAP2C | chr7:100769550-100770021 | enh_46964 | 1D-eRNA |
| TFAP2C | chr7:130698176-130698385 | enh_47408 | 1D-eRNA |
| TFAP2C | chr8:17323878-17324161 | enh_48028 | 1D-eRNA |
| TFAP2C | chr8:134418913-134419546 | enh_50384 | 1D-eRNA |
| TFAP2C | chr8:144097339-144097994 | enh_50608 | 1D-eRNA |
| TFAP2C | chr9:91147215-91147519 | enh_51632 | 1D-eRNA |
| TFAP2C | chr9:93763437-93763900 | enh_51796 | 1D-eRNA |
| TFAP2C | chr9:124132405-124132992 | enh_52613 | 1D-eRNA |
| TFAP2C | chr9:132319647-132320020 | enh_52882 | 1D-eRNA |
| TFAP2C | chrX:41240919-41241336 | enh_53489 | 1D-eRNA |
| TFAP2C | chr1:10488077-10488444 | enh_319 | no-eRNA |
| TFAP2C | chr1:16125532-16125808 | enh_453 | no-eRNA |
| TFAP2C | chr1:16126596-16127054 | enh_455 | no-eRNA |
| TFAP2C | chr1:16403421-16403745 | enh_466 | no-eRNA |
| TFAP2C | chr1:16515501-16515794 | enh_490 | no-eRNA |
| TFAP2C | chr1:25900092-25900426 | enh_795 | no-eRNA |
| TFAP2C | chr1:36952509-36953025 | enh_1167 | no-eRNA |
| TFAP2C | chr1:41328195-41328732 | enh_1272 | no-eRNA |
| TFAP2C | chr1:41410021-41410320 | enh_1288 | no-eRNA |
| TFAP2C | chr1:95417136-95417445 | enh_2398 | no-eRNA |
| TFAP2C | chr1:101723491-101723769 | enh_2485 | no-eRNA |
| TFAP2C | chr1:110334803-110335456 | enh_2525 | no-eRNA |
| TFAP2C | chr1:116832866-116833310 | enh_2760 | no-eRNA |
| TFAP2C | chr1:117030712-117030975 | enh_2786 | no-eRNA |
| TFAP2C | chr1:153328065-153328260 | enh_3132 | no-eRNA |
| TFAP2C | chr1:173092146-173092533 | enh_3622 | no-eRNA |
| TFAP2C | chr1:181059027-181059609 | enh_3768 | no-eRNA |
| TFAP2C | chr1:202075385-202075885 | enh_4186 | no-eRNA |
| TFAP2C | chr1:202076371-202076691 | enh_4186 | no-eRNA |
| TFAP2C | chr1:202077431-202077775 | enh_4187 | no-eRNA |
| TFAP2C | chr1:207414797-207415137 | enh_4407 | no-eRNA |
| TFAP2C | chr1:211811951-211812530 | enh_4552 | no-eRNA |
| TFAP2C | chr1:212102306-212102926 | enh_4579 | no-eRNA |
| TFAP2C | chr1:225924883-225925193 | enh_4983 | no-eRNA |
| TFAP2C | chr1:230960820-230961127 | enh_5205 | no-eRNA |
| TFAP2C | chr1:234700447-234700681 | enh_5316 | no-eRNA |
| TFAP2C | chr1:234793901-234794235 | enh_5354 | no-eRNA |
| TFAP2C | chr1:234859848-234860256 | enh_5378 | no-eRNA |
| TFAP2C | chr10:4889985-4890228 | enh_5865 | no-eRNA |
| TFAP2C | chr10:12306309-12306570 | enh_6100 | no-eRNA |
| TFAP2C | chr10:12375797-12376409 | enh_6104 | no-eRNA |
| TFAP2C | chr10:21653356-21653687 | enh_6165 | no-eRNA |
| TFAP2C | chr10:32229828-32230072 | enh_6492 | no-eRNA |
| TFAP2C | chr10:75647399-75647997 | enh_7199 | no-eRNA |
| TFAP2C | chr10:93392822-93393211 | enh_7679 | no-eRNA |
| TFAP2C | chr10:112216372-112216929 | enh_7978 | no-eRNA |
| TFAP2C | chr10:116484060-116484426 | enh_8104 | no-eRNA |
| TFAP2C | chr11:2962348-2962699 | enh_8547 | no-eRNA |
| TFAP2C | chr11:47211116-47211268 | enh_9220 | no-eRNA |
| TFAP2C | chr11:47959563-47960175 | enh_9248 | no-eRNA |
| TFAP2C | chr11:65259323-65259506 | enh_9542 | no-eRNA |
| TFAP2C | chr11:67159184-67159365 | enh_9623 | no-eRNA |
| TFAP2C | chr11:67913587-67913810 | enh_9648 | no-eRNA |
| TFAP2C | chr11:75981504-75982103 | enh_9905 | no-eRNA |
| TFAP2C | chr11:76481999-76482325 | enh_9948 | no-eRNA |
| TFAP2C | chr11:85885127-85885790 | enh_10024 | no-eRNA |
| TFAP2C | chr11:112192606-112192854 | enh_10422 | no-eRNA |
| TFAP2C | chr12:46867482-46867774 | enh_11680 | no-eRNA |
| TFAP2C | chr12:54219202-54219491 | enh_11957 | no-eRNA |
| TFAP2C | chr12:76371649-76372265 | enh_12432 | no-eRNA |
| TFAP2C | chr12:116844330-116844738 | enh_13295 | no-eRNA |
| TFAP2C | chr12:121123920-121124638 | enh_13380 | no-eRNA |
| TFAP2C | chr12:122064311-122064949 | enh_13402 | no-eRNA |
| TFAP2C | chr12:125211734-125212226 | enh_13485 | no-eRNA |
| TFAP2C | chr12:132345256-132345788 | enh_13619 | no-eRNA |
| TFAP2C | chr13:95966744-95967081 | enh_14768 | no-eRNA |
| TFAP2C | chr13:99325492-99325645 | enh_14812 | no-eRNA |
| TFAP2C | chr13:110651106-110651477 | enh_14950 | no-eRNA |
| TFAP2C | chr13:114992230-114992393 | enh_15079 | no-eRNA |
| TFAP2C | chr14:35806010-35806347 | enh_15307 | no-eRNA |
| TFAP2C | chr14:71585147-71585753 | enh_15908 | no-eRNA |
| TFAP2C | chr14:74262763-74263030 | enh_15948 | no-eRNA |
| TFAP2C | chr14:75866986-75867296 | enh_16038 | no-eRNA |
| TFAP2C | chr14:77419695-77420183 | enh_16091 | no-eRNA |
| TFAP2C | chr14:91884833-91885047 | enh_16264 | no-eRNA |
| TFAP2C | chr14:93596739-93597316 | enh_16284 | no-eRNA |
| TFAP2C | chr14:96562248-96562600 | enh_16372 | no-eRNA |
| TFAP2C | chr14:99849436-99850078 | enh_16413 | no-eRNA |
| TFAP2C | chr14:102172594-102172839 | enh_16519 | no-eRNA |
| TFAP2C | chr14:103720204-103720431 | enh_16577 | no-eRNA |
| TFAP2C | chr15:31588917-31589223 | enh_16755 | no-eRNA |
| TFAP2C | chr15:35014232-35014868 | enh_16782 | no-eRNA |
| TFAP2C | chr15:40346965-40347328 | enh_17001 | no-eRNA |
| TFAP2C | chr15:41198213-41198878 | enh_17033 | no-eRNA |
| TFAP2C | chr15:59981679-59981811 | enh_17267 | no-eRNA |
| TFAP2C | chr15:60383389-60383607 | enh_17273 | no-eRNA |
| TFAP2C | chr15:63189241-63189820 | enh_17334 | no-eRNA |
| TFAP2C | chr15:63741892-63742161 | enh_17379 | no-eRNA |
| TFAP2C | chr15:67316741-67317023 | enh_17487 | no-eRNA |
| TFAP2C | chr15:67342080-67342360 | enh_17497 | no-eRNA |
| TFAP2C | chr15:69425875-69426071 | enh_17563 | no-eRNA |
| TFAP2C | chr15:70488745-70489302 | enh_17635 | no-eRNA |
| TFAP2C | chr15:70490085-70490682 | enh_17637 | no-eRNA |
| TFAP2C | chr15:70730203-70730573 | enh_17669 | no-eRNA |
| TFAP2C | chr15:77789555-77789864 | enh_17861 | no-eRNA |
| TFAP2C | chr15:85144054-85144774 | enh_17976 | no-eRNA |
| TFAP2C | chr16:73158257-73158867 | enh_19400 | no-eRNA |
| TFAP2C | chr16:81437166-81437388 | enh_19521 | no-eRNA |
| TFAP2C | chr16:85384605-85384844 | enh_19691 | no-eRNA |
| TFAP2C | chr16:85548382-85549087 | enh_19740 | no-eRNA |
| TFAP2C | chr16:87823502-87823928 | enh_19886 | no-eRNA |
| TFAP2C | chr17:4848581-4849168 | enh_20058 | no-eRNA |
| TFAP2C | chr17:40819480-40820112 | enh_20819 | no-eRNA |
| TFAP2C | chr17:41535228-41535577 | enh_20872 | no-eRNA |
| TFAP2C | chr17:49029387-49029714 | enh_21234 | no-eRNA |
| TFAP2C | chr18:3013333-3013609 | enh_22142 | no-eRNA |
| TFAP2C | chr18:19693462-19693717 | enh_22336 | no-eRNA |
| TFAP2C | chr19:13273489-13274169 | enh_23503 | no-eRNA |
| TFAP2C | chr19:18411973-18412605 | enh_23662 | no-eRNA |
| TFAP2C | chr19:18414208-18414497 | enh_23662 | no-eRNA |
| TFAP2C | chr19:19174189-19174735 | enh_23694 | no-eRNA |
| TFAP2C | chr19:41426818-41427254 | enh_23927 | no-eRNA |
| TFAP2C | chr2:10592567-10592862 | enh_24612 | no-eRNA |
| TFAP2C | chr2:11530104-11530425 | enh_24640 | no-eRNA |
| TFAP2C | chr2:20732831-20733540 | enh_24960 | no-eRNA |
| TFAP2C | chr2:26958113-26958424 | enh_25079 | no-eRNA |
| TFAP2C | chr2:28583257-28583591 | enh_25135 | no-eRNA |
| TFAP2C | chr2:42190697-42191218 | enh_25519 | no-eRNA |
| TFAP2C | chr2:42193602-42194051 | enh_25523 | no-eRNA |
| TFAP2C | chr2:43383050-43383277 | enh_25684 | no-eRNA |
| TFAP2C | chr2:48334848-48335076 | enh_25855 | no-eRNA |
| TFAP2C | chr2:113402952-113403495 | enh_27100 | no-eRNA |
| TFAP2C | chr2:127928091-127928381 | enh_27351 | no-eRNA |
| TFAP2C | chr2:234259434-234259685 | enh_29238 | no-eRNA |
| TFAP2C | chr2:241308121-241308734 | enh_29621 | no-eRNA |
| TFAP2C | chr20:3800748-3801286 | enh_29784 | no-eRNA |
| TFAP2C | chr20:30181109-30181903 | enh_30308 | no-eRNA |
| TFAP2C | chr20:30183420-30183966 | enh_30308 | no-eRNA |
| TFAP2C | chr20:30198514-30198914 | enh_30315 | no-eRNA |
| TFAP2C | chr20:32902447-32902652 | enh_30377 | no-eRNA |
| TFAP2C | chr20:46082129-46082437 | enh_30650 | no-eRNA |
| TFAP2C | chr20:48232220-48232673 | enh_30732 | no-eRNA |
| TFAP2C | chr21:16582467-16582848 | enh_31301 | no-eRNA |
| TFAP2C | chr21:34752889-34753185 | enh_31530 | no-eRNA |
| TFAP2C | chr21:40169512-40169761 | enh_31678 | no-eRNA |
| TFAP2C | chr21:44465551-44465819 | enh_31864 | no-eRNA |
| TFAP2C | chr21:44465822-44466069 | enh_31864 | no-eRNA |
| TFAP2C | chr21:46750107-46750683 | enh_32066 | no-eRNA |
| TFAP2C | chr3:9005053-9005204 | enh_33188 | no-eRNA |
| TFAP2C | chr3:49169721-49170026 | enh_33983 | no-eRNA |
| TFAP2C | chr3:49577344-49578059 | enh_34005 | no-eRNA |
| TFAP2C | chr3:122233408-122233999 | enh_34883 | no-eRNA |
| TFAP2C | chr3:189949860-189950124 | enh_36107 | no-eRNA |
| TFAP2C | chr3:193721183-193721661 | enh_36161 | no-eRNA |
| TFAP2C | chr3:194053928-194054762 | enh_36237 | no-eRNA |
| TFAP2C | chr3:194751433-194751977 | enh_36304 | no-eRNA |
| TFAP2C | chr3:194754092-194754608 | enh_36304 | no-eRNA |
| TFAP2C | chr3:195862843-195863356 | enh_36337 | no-eRNA |
| TFAP2C | chr3:197217024-197217651 | enh_36389 | no-eRNA |
| TFAP2C | chr4:38320540-38321151 | enh_36986 | no-eRNA |
| TFAP2C | chr4:40397953-40398186 | enh_37062 | no-eRNA |
| TFAP2C | chr4:74975231-74975567 | enh_37264 | no-eRNA |
| TFAP2C | chr4:78077646-78078268 | enh_37308 | no-eRNA |
| TFAP2C | chr5:15008744-15008914 | enh_38729 | no-eRNA |
| TFAP2C | chr5:33148408-33148641 | enh_38799 | no-eRNA |
| TFAP2C | chr5:43432613-43432848 | enh_39041 | no-eRNA |
| TFAP2C | chr5:54038212-54038675 | enh_39141 | no-eRNA |
| TFAP2C | chr5:133802152-133802641 | enh_40687 | no-eRNA |
| TFAP2C | chr5:149730115-149730375 | enh_41232 | no-eRNA |
| TFAP2C | chr5:168027407-168027844 | enh_41486 | no-eRNA |
| TFAP2C | chr5:172141775-172142323 | enh_41591 | no-eRNA |
| TFAP2C | chr5:173221147-173221523 | enh_41729 | no-eRNA |
| TFAP2C | chr5:176882405-176882730 | enh_41807 | no-eRNA |
| TFAP2C | chr6:16965028-16965346 | enh_42520 | no-eRNA |
| TFAP2C | chr6:26553976-26554272 | enh_42759 | no-eRNA |
| TFAP2C | chr6:33807870-33808539 | enh_42939 | no-eRNA |
| TFAP2C | chr6:37483660-37484375 | enh_43073 | no-eRNA |
| TFAP2C | chr6:52783052-52783485 | enh_43481 | no-eRNA |
| TFAP2C | chr6:82476966-82477245 | enh_43716 | no-eRNA |
| TFAP2C | chr7:27702595-27702925 | enh_46028 | no-eRNA |
| TFAP2C | chr7:106209013-106209662 | enh_47088 | no-eRNA |
| TFAP2C | chr7:155711725-155712371 | enh_47778 | no-eRNA |
| TFAP2C | chr8:8458359-8458666 | enh_47907 | no-eRNA |
| TFAP2C | chr8:40032245-40032889 | enh_48581 | no-eRNA |
| TFAP2C | chr8:109152119-109152500 | enh_49593 | no-eRNA |
| TFAP2C | chr8:125212383-125212941 | enh_49896 | no-eRNA |
| TFAP2C | chr8:126464241-126464702 | enh_49948 | no-eRNA |
| TFAP2C | chr8:126559339-126559690 | enh_49976 | no-eRNA |
| TFAP2C | chr8:126568623-126568919 | enh_49979 | no-eRNA |
| TFAP2C | chr8:126656659-126657011 | enh_50002 | no-eRNA |
| TFAP2C | chr8:143807795-143808411 | enh_50589 | no-eRNA |
| TFAP2C | chr9:33415586-33415945 | enh_51060 | no-eRNA |
| TFAP2C | chr9:86577128-86577440 | enh_51455 | no-eRNA |
| TFAP2C | chr9:89987451-89987723 | enh_51591 | no-eRNA |
| TFAP2C | chr9:123698863-123699098 | enh_52602 | no-eRNA |

**Supplementary Table 2** TFs and regulated enhancers

| **Characteristics** | **χ^2^** | ***P*** |
| --- | --- | --- |
| DAPK1 | 0.1437 | 0.7046 |
| Gender | 0.43365 | 0.5102 |
| Age | 0.39993 | 0.5271 |
| Child-Pugh grade | 0.0030181 | 0.9562 |
| GLOBAL | 1.891 | 0.7558 |

**Supplementary Table 3** VIF assumptions

| Characteristics | VIF |
| --- | --- |
| DAPK1 | 1.0889 |
| Gender |  |
| Female | Reference |
| Male | 1.0801 |
| Age |  |
| <= 60 | Reference |
| > 60 | 1.1268 |
| Child-Pugh grade |  |
| A | Reference |
| B&C | 1.0666 |

**Supplementary Table 4** The infiltration landscape of DAPK1 and immune cells in HCC

|  | Cell | Pearson | P Value (Pearson) | Spearman | P Value (Spearman) |
| --- | --- | --- | --- | --- | --- |
| DAPK1 | aDC | 0.068 | 0.191 | 0.031 | 0.548 |
| DAPK1 | B cells | 0.037 | 0.479 | 0.022 | 0.670 |
| DAPK1 | CD8 T cells | 0.106 | 0.041 | 0.080 | 0.123 |
| DAPK1 | Cytotoxic cells | -0.163 | 0.002 | -0.177 | <0.001 |
| DAPK1 | DC | -0.179 | <0.001 | -0.190 | <0.001 |
| DAPK1 | Eosinophils | 0.133 | 0.010 | 0.142 | 0.006 |
| DAPK1 | iDC | 0.020 | 0.707 | -0.021 | 0.679 |
| DAPK1 | Macrophages | 0.053 | 0.307 | 0.026 | 0.618 |
| DAPK1 | Mast cells | 0.127 | 0.014 | 0.120 | 0.020 |
| DAPK1 | Neutrophils | -0.071 | 0.170 | -0.104 | 0.044 |
| DAPK1 | NK CD56bright cells | 0.037 | 0.473 | 0.019 | 0.715 |
| DAPK1 | NK CD56dim cells | 0.059 | 0.255 | 0.014 | 0.787 |
| DAPK1 | NK cells | 0.260 | <0.001 | 0.212 | <0.001 |
| DAPK1 | pDC | -0.208 | <0.001 | -0.218 | <0.001 |
| DAPK1 | T cells | 0.027 | 0.609 | 0.015 | 0.771 |
| DAPK1 | T helper cells | 0.303 | <0.001 | 0.282 | <0.001 |
| DAPK1 | Tcm | 0.191 | <0.001 | 0.193 | <0.001 |
| DAPK1 | Tem | 0.135 | 0.009 | 0.105 | 0.043 |
| DAPK1 | TFH | 0.172 | <0.001 | 0.152 | 0.003 |
| DAPK1 | Tgd | -0.010 | 0.849 | -0.029 | 0.573 |
| DAPK1 | Th1 cells | 0.070 | 0.175 | 0.029 | 0.582 |
| DAPK1 | Th17 cells | 0.028 | 0.590 | 0.054 | 0.296 |
| DAPK1 | Th2 cells | 0.246 | <0.001 | 0.246 | <0.001 |
| DAPK1 | TReg | -0.091 | 0.079 | -0.105 | 0.042 |
